# Body mass index, subjective body shape, and suicidal ideation among community-dwelling Korean adults

**DOI:** 10.1186/s13690-021-00627-y

**Published:** 2021-06-08

**Authors:** Chae Eun Yong, Young Bum Kim, Jiyoung Lyu

**Affiliations:** 1 Interdisciplinary Program of Studies of Life Education, Hallym University, Chuncheon, South Korea; 2 Institute of Aging, Hallym University, Chuncheon, South Korea

**Keywords:** Body mass index, Subjective body shape, Suicidal ideation, Developmental stages, KCHS

## Abstract

**Background:**

Previous studies have not investigated in depth the combination of objective body weight and subjective body shape and its association with suicidal ideation among different age groups. Therefore, this study aimed to examine the abovementioned association among Korean adults, stratified by developmental stages.

**Methods:**

We used nationally representative data from the 2017 Korean Community Health Survey, and included 222,037 participants aged 19 years or older in our study. Suicidal ideation was considered as the dependent variable (1 = yes, 0 = no). Along with body mass index (BMI) and subjective body shape measures, seven categories were created (1 = underweight-skinny, 2 = underweight-normal/fat, 3 = healthy weight-skinny, 4 = healthy weight-normal, 5 = healthy weight-fat, 6 = overweight-skinny/normal, 7 = overweight-fat). Multivariate logistic regression was conducted for each age group.

**Results:**

Adjusted for covariates, young adults who were overweight-fat (OR = 1.18, *p* < .01), middle-aged adults who were underweight-skinny (OR = 1.32, *p* < .05), and older adults who were healthy weight-fat (OR = 1.19, *p* < .05) were more likely to have suicidal ideation than their healthy weight-normal counterparts.

**Conclusions:**

The results suggest that the association between the combination of objective body weight and subjective body shape and suicidal ideation differs according to the developmental stage. Therefore, this difference should be considered when developing suicide prevention interventions based on the developmental stages.

## Background

Among the Organisation for Economic Co-operation and Development (OECD) countries, Korea has been noted as having the highest suicide rates between 2003 and 2016 [1]. In 2018, 26.6 persons out of 100,000 Koreans died by suicide, and this rate was twice the average of the OECD countries [1]. Therefore, studies identifying the risk factors for suicide have been conducted widely among Koreans.

Previous studies have shown that body mass index (BMI) was associated with suicidal risk [2–4]. Overall, overweight (BMI ≥ 25.0 kg/m^2^) people were more likely to have depression, high stress level, and suicidal ideation than healthy weight (18.5 ≤ BMI < 25.0 kg/m^2^) people [2, 5–7]. In addition, several studies have shown that being underweight (BMI < 18.5 kg/m^2^) was associated with a high risk of suicidal ideation [8–10].

Apart from body weight, some researchers have found that perceived body shape was a risk factor for mental disorders [11–15]. When adolescents perceived their body as skinny or fat, their stress and depression levels increased [16]. In addition, when college students were more satisfied with their body image, their subjective well-being was higher [17]. Similarly, older adults with a positive perceived body shape had lower levels of anxiety and depression [18].

Taking both objective body weight and subjective body shape into account, several studies have examined the association between body shape discordance and mental health outcomes [14, 15, 19–21]. When objective body weight and subjective body shape were not consistent, people were more likely to have depression and suicidal ideation. According to Seo et al. [22], young men who perceived themselves as fatter than their actual body weight and middle-aged men who perceived themselves as slimmer than their actual body weight had the highest depression level. Moreover, Shin et al. [23] found that people who were actually underweight but perceived themselves as fatter were more likely to have depression than those who were actually overweight but perceived themselves as slimmer. Contrary to the finding that body shape discordance was harmful for mental health, some study findings have shown that people had a low quality of life or depression even when actual body weight and subjective body shape matched (i.e., underweight-skinny or overweight-fat) [24, 25].

However, the ideal body shape standard may differ depending on the developmental stage. Generally, younger people are more interested in their appearance and are more sensitive to their subjective body shape than older people [26]. For example, young adults prefer a skinny body shape due to the increase in social media use, and often stigmatize themselves as “a loser” when they perceive themselves as fat [27]. In contrast, older adults become concerned about their health when they lose weight for no reason [28].

Although existing studies have revealed an association between body mass index, subjective body shape, and mental health outcomes, most studies were limited to adolescents or younger age groups; therefore, it was difficult to compare the abovementioned association by different age groups. Therefore, this study aimed to examine the association between body mass index, subjective body shape, and suicidal ideation among Korean adults using the 2017 Korean Community Health Survey. More specifically, this study examined the above association according to different age groups: young adults (age 19–44 years), middle-aged adults (age 45–64 years), and older adults (age 65+).

## Methods

### Data

Data for this study were obtained from the 2017 Korean Community Health Survey (KCHS), a nationally representative survey conducted by the Korea Centers for Disease Control and Prevention (http://chs.cdc.go.kr). The study sample was selected with a complex sampling design, first stratified by 253 communities in which the health centers were located. Then, the type of residence (apartment or house) was sampled via probability proportional to the size of county units. The surveyed population was limited to residents aged 19 years or older. This community-based survey included information about socio-demographics, health status, and healthcare utilization. The KCHS was administered through computer-assisted personal interviewing (CAPI) conducted by trained face-to-face interviewers [29, 30]. In 2017, 228,381 adults from 253 communities completed the interviews. After listwise deletion of cases with missing values, 222,037 adults remained for the empirical analyses. To compare the age group differences, the sample was divided into young adults (age 19–44 years; *N* = 99,831), middle-aged adults (age 45–64 years; *N* = 84,913), and older adults (age 65+; *N* = 37,293).

### Measures

#### Suicidal ideation

Suicidal ideation was measured based on the question, “During the last 12 months, did you ever think about suicide?” The response was dichotomous (1 = yes, 0 = no) [31].

#### Body shape categories

Body shape categories were measured using body mass index (BMI) and subjective body shape. First, based on the BMI (body weight divided by the height squared) classification [32], three categories were determined: (a) underweight (BMI < 18.5 kg/m^2^), (b) healthy weight (18.5 ≤ BMI < 25.0 kg/m^2^), and (c) overweight (BMI ≥ 25.0 kg/m^2^). Second, subjective body shape was assessed with five response options (1 = very skinny, 2 = skinny, 3 = normal, 4 = fat, 5 = very fat), and to make consistent categories between BMI and subjective body shape measures, three categories were determined: (a) skinny (very skinny/skinny), (b) normal (normal), and (c) fat (fat/very fat). Finally, using both BMI and subjective body shape measures, seven categories were created to show whether respondents had a right perception, underestimation, or overestimation. For example, if respondents were in the underweight category but perceived their body shape as normal or fat, it was considered overestimation (1 = underweight-skinny, 2 = underweight-normal/fat, 3 = healthy weight-skinny, 4 = healthy weight-normal (reference), 5 = healthy weight-fat, 6 = overweight-skinny/normal, 7 = overweight-fat) [25].

#### Covariates

In this study, sex (1 = female, 0 = male), age (number of years), education (less than elementary school graduate, middle school graduate, high school graduate, college or more [reference group]), monthly household income (<$1000, <$2000, <$3000, <$4000, <$5000, <$6000, ≥$6000 [reference group]), living alone (1 = yes, 0 = no) [33], and economic activity (1 = yes, 0 = no) [33] were included as sociodemographic covariates.

Several health-related covariates were also included in this study. Self-rated health was dichotomized as (1 = fair/poor, 0 = excellent/very good/good). Depression was measured using the Patient Health Questionnaire-9 (PHQ-9) [34]. The PHQ-9 score ranged from 0 to 27, and the Cronbach’s alpha was .814. Current drinking status (1 = yes, 0 = no) [35] and current smoking status (1 = yes, 0 = no) [35] were also included. If respondents tried to lose weight or keep themselves from gaining, weight control practice was dichotomized as (1 = yes, 0 = no). If respondents had at least 20 min of vigorous activity more than three times a week, or had at least 30 min of moderate activity more than five times a week, physical activity was dichotomized as (1 = yes, 0 = no).

### Statistical analysis

In this study, analyses were conducted separately for each age group (young adults, middle-aged adults, and older adults). To present sample characteristics, means (M) and standard deviations (SD) for continuous variables, and weighted percentages (W%) for categorical variables were provided as descriptive statistics. One-way ANOVA and chi-square tests were conducted to evaluate age group differences as descriptive statistics. Then, a multivariate logistic regression was used to investigate the association between body shape categories and suicidal ideation while simultaneously controlling for covariates. All statistical analyses were based on weighted data, which accounted for the complex sample survey design, using SPSS version 21.

## Results

### Descriptive statistics

Table 1 presents the descriptive statistics for the study sample. To examine age group differences, participants were divided into three groups based on developmental stages (young adults: 19–44 years old, middle-aged adults: 45–64 years old, and older adults: 65 years and over). Suicidal ideation was most prevalent among older adults (11.0%), compared to young adults (5.3%). As for body shape categories, the majority of study participants were in the healthy weight-normal category, and a minority of them were in the underweight-normal/fat category in all age groups. All study variables were significantly different according to age group.

**Table 1:** Sample characteristics by age group from 2017 Korean Community Health Survey

Variables	M(SD)/W%				*P*-value
	All (*N* = 222,037)	Young adults (*N* = 99,831)	Middle-aged adults (*N* = 84,913)	Older adults (*N* = 37,293)	
Suicidal ideation (yes)	6.8%	5.3%	6.7%	11.0%	***
BMI (kg/m^2^)	23.4 (3.3)	23.1 (3.7)	23.7 (2.9)	23.3 (3.0)	***
Body shape discordance					***
Underweight-skinny	3.7%	5.0%	1.8%	4.3%	
Underweight-normal/fat	1.2%	2.0%	0.4%	0.7%	
Healthyweight-skinny	12.2%	10.9%	11.0%	18.6%	
Healthyweight-normal (ref.)	38.8%	37.6%	39.9%	39.7%	
Healthyweight-fat	16.5%	17.8%	17.5%	10.6%	
Overweight-skinny/normal	4.7%	3.0%	5.3%	7.5%	
Overweight-fat	22.9%	23.6%	24.0%	18.7%	
Sex (female)	49.9%	48.0%	50.0%	54.3%	***
Education					***
Less than elementary school graduate	12.3%	0.2%	8.9%	52.0%	
Middle school graduate	8.4%	1.2%	12.9%	17.2%	
High school graduate	37.6%	39.4%	43.0%	20.3%	
College and more (ref.)	41.8%	59.2%	35.2%	10.5%	
Monthly household income					***
< $1000	10.3%	3.2%	6.4%	38.2%	
< $2000	12.1%	7.7%	12.0%	24.4%	
< $3000	17.1%	18.1%	16.9%	15.3%	
< $4000	18.0%	21.5%	18.0%	8.8%	
< $5000	15.2%	18.2%	16.1%	5.1%	
< $6000	10.1%	12.1%	10.9%	2.9%	
≥ $6000 (ref.)	17.1%	19.2%	19.8%	5.4%	
Living alone (yes)	10.0%	8.1%	8.1%	19.0%	***
Economic activity (yes)	65.1%	69.8%	75.0%	29.7%	***
Self-rated health (fair/poor)	14.4%	5.3%	13.8%	39.8%	***
Depression (0–27)	2.1 (0.0)	2.1 (0.0)	1.8 (0.0)	2.7 (0.0)	***
Current drinking status (yes)	76.1%	87.0%	76.1%	46.6%	***
Current smoking status (yes)	20.4%	23.8%	21.3%	9.3%	***
Weight control practice (yes)	68.3%	75.5%	68.7%	48.0%	***
Physical activity (yes)	22.4%	23.4%	23.5%	17.1%	***

*M Mean, SD Standard Deviation, W%* Weighted %, *p-value* ANOVA or x^2^ test

*** *P* < .001

### Logistic regression results

Table 2 shows the multivariate logistic regression results by age group. Among young adults, respondents in the overweight-fat category were more likely to have suicidal ideation than respondents in the healthy weight-normal category (OR = 1.18, *p* < .01). Among middle-aged adults, respondents in the underweight-skinny category were more likely to have suicidal ideation than respondents in the healthy weight-normal category (OR = 1.32, *p* < .05). The odds of having suicidal ideation was 19% higher for older adults in the healthy weight-fat category than in older adults in the healthy weight-normal category (OR = 1.19, *p* < .05).

**Table 2:** The odds ratio (OR) of suicidal ideation in relation to body mass index, and subjective body shape. Results from multivariate logistic regression. Korean Community Health Survey 2017

Variables	Suicidal ideation, OR(95% CI)			
	All (*N* = 222,037)	Young adults (*N* = 99,831)	Middle-aged adults (*N* = 84,913)	Older adults (*N* = 37,293)
Body shape discordance (Healthyweight-normal = ref.)				
Underweight-skinny	1.16 (1.03, 1.31)*	1.19 (0.98, 1.45)	1.32 (1.02, 1.72)*	1.16 (0.97, 1.40)
Underweight-normal/fat	1.10 (0.89, 1.37)	1.00 (0.73, 1.36)	1.32 (0.88, 2.00)	1.26 (0.86, 1.85)
Healthyweight-skinny	1.06 (0.98, 1.14)	1.14 (0.97, 1.34)	1.07 (0.94, 1.22)	1.10 (0.98, 1.24)
Healthyweight-fat	1.12 (1.04, 1.20)**	1.11 (0.98, 1.25)	1.04 (0.92, 1.22)	1.19 (1.04, 1.37)*
Overweight-skinny/normal	0.97 (0.85, 1.11)	0.69 (0.47, 1.02)	0.93 (0.74, 1.16)	1.14 (0.96, 1.36)
Overweight-fat	1.14 (1.06, 1.22)***	1.18 (1.05, 1.33)**	1.08 (0.97, 1.21)	1.10 (0.97, 1.24)
Sex (female)	1.40 (1.32, 1.49)***	1.60 (1.43, 1.79)***	1.28 (1.15, 1.42)***	1.14 (1.03, 1.26)*
Age (years)	1.01 (1.00, 1.01)***	1.03 (1.02, 1.04)***	0.99 (0.98, 1.00)*	1.00 (0.99, 1.01)
Education (College and more = ref.)				
Less than elementary school graduate	1.38 (1.25, 1.53)***	3.38 (1.92, 5.92)***	1.70 (1.45, 1.99)***	1.46 (1.20, 1.79)***
Middle school graduate	1.41 (1.28, 1.55)***	2.62 (1.96, 3.51)***	1.26 (1.09, 1.46)**	1.35 (1.08, 1.69)**
High school graduate	1.30 (1.21, 1.38)***	1.39 (1.26, 1.53)***	1.23 (1.10, 1.37)***	1.20 (0.97, 1.50)
Monthly household income (≥$6000 = ref.)				
< $1000	1.95 (1.74, 2.19)***	2.23 (1.77, 2.82)***	2.30 (1.91, 2.76)***	1.15 (0.90, 1.46)
< $2000	1.61 (1.44, 1.79)***	1.65 (1.37, 1.98)***	1.96 (1.67, 2.31)***	0.80 (0.62, 1.03)
< $3000	1.44 (1.30, 1.59)***	1.53 (1.30, 1.79)***	1.54 (1.32, 1.80)***	0.79 (0.60, 1.03)
< $4000	1.22 (1.10, 1.36)***	1.22 (1.04, 1.42)*	1.31 (1.11, 1.54)**	0.74 (0.55, 0.99)*
< $5000	1.10 (0.99, 1.23)	1.11 (0.94, 1.31)	1.15 (0.97, 1.36)	0.79 (0.57, 1.09)
< $6000	1.15 (1.01, 1.30)*	1.15 (0.96, 1.37)	1.17 (0.96, 1.43)	1.01 (0.71, 1.45)
Living alone (yes)	1.03 (0.95, 1.10)	0.97 (0.83, 1.13)	1.17 (1.04, 1.32)**	1.05 (0.94, 1.17)
Economic activity (yes)	0.96 (0.91, 1.02)	0.85 (0.77, 0.94)**	0.97 (0.88, 1.06)	0.88 (0.80, 0.97)*
Self-rated health (fair/poor)	1.69 (1.59, 1.80)***	1.78 (1.55, 2.04)***	1.75 (1.59, 1.93)***	1.63 (1.48, 1.79)***
Depression (0–27)	1.34 (1.33, 1.35)***	1.37 (1.35, 1.39)***	1.38 (1.36, 1.40)***	1.28 (1.26, 1.29)***
Current drinking status (yes)	1.21 (1.14, 1.29)***	1.09 (0.95, 1.24)	1.23 (1.12, 1.35)***	1.12 (1.03, 1.23)*
Current smoking status (yes)	1.33 (1.25, 1.44)***	1.15 (1.02, 1.31)*	1.34 (1.19, 1.51)***	1.39 (1.21, 1.60)***
Weight control practice (yes)	1.09 (1.03, 1.15)**	1.11 (0.99, 1.24)	1.04 (0.95, 1.14)	1.09 (1.00, 1.19)
Physical activity (yes)	1.05 (0.99, 1.12)	1.06 (0.95, 1.17)	1.05 (0.95, 1.16)	1.03 (0.91, 1.17)
Nagelkerke *R*^2^	.298	.296	.300	.290

*OR* Odds ratio, *CI* Confidence interval

* *P* < .05, **P < .01, *** *P* < .001

As for covariates, sex, education, self-rated health, depression, and smoking status were significantly associated with suicidal ideation in all age groups.

## Discussion

This study aimed to examine the association between body mass index, subjective body shape, and suicidal ideation among Korean adults by age group. Adjusted for covariates, results showed that the combination of objective body weight and subjective body shape and its association with suicidal ideation differed by age group.

Among young adults (age 19–44 years), those in the overweight-fat category were more likely to have suicidal ideation. This result was consistent with previous findings in a relatively young population [24, 36, 37]. According to Yazdani et al. [37], overweight and obese people were more likely to be dissatisfied with their body image, which could have negative effects on psychological well-being. In addition, negative social gaze on obese individuals can lead to high stress levels, which is likely to accompany suicidal behaviors [36].

Among middle-aged adults (age 45–64 years), those in the underweight-skinny category were more likely to have suicidal ideation. This result was similar to findings that people who were underweight or perceived themselves as skinny had worse mental health than healthy weight people [8, 12, 22, 38]. According to Coin et al. [39], malnutrition was often associated with being underweight, resulting in decreased immunity, anemia, osteoporosis, and muscle loss. Since Korean middle-aged adults are from the baby boomer generation who may have previously experienced poverty and lived with insufficient food, they might be susceptible to the above risks. Therefore, middle-aged adults who are underweight or perceive themselves as skinny might have a low level of health-related quality of life, which is associated with suicidal ideation [40].

Among older adults (age 65+), those in the healthy weight-fat category were more likely to have suicidal ideation. Indeed, obesity in the elderly is a known risk factor for cardiovascular disease, cancer, and mortality [41]. As people age, their health concerns increase. Therefore, even perceiving themselves as fat can lead them to have health concerns and a poor quality of life, which can be associated with suicidal ideation.

The findings suggest that not only people with a discrepancy between objective body weight and subjective body perception (i.e., healthy weight-fat category), but those outside the normal range of body weight defined by the society (i.e., underweight-skinny, overweight-fat) also are at suicidal risk. In other words, people who would like to be in the normal category but are actually in the abnormal category may be frustrated when there is a disparity between their needs. Although some tendencies might differ by age and gender, this is in line with Etcoff’s [42] finding that normal body shape was preferred over very skinny or very fat body shape because of physical attractiveness.

In socio-cultural contexts, people form their own values and norms about body shapes, and individual characteristics such as self-esteem can be associated with them [43]. Nevertheless, negative views or perceptions about the body shape caused by lookism in Korean society [44] result in negative emotional reactions such as sadness and loneliness when their needs have discrepancies. When people have low self-esteem, they feel worthless, which makes it difficult to have rational thoughts [44]. In sum, elevating self-esteem by relieving negative thoughts and escaping from sociocultural pressure about body shapes is required.

There are some limitations to this study that should be noted. First, suicidal ideation was measured with a single question; therefore, the level of suicidal risk (severity) could not be identified in detail. Second, when determining body shape categories, the sample size was very small in the underweight-normal/fat group. Therefore, these results should be interpreted with caution. Lastly, the 2017 KCHS is a cross-sectional data collection survey; therefore, the causal relationship could not be explored.

Although this study has several limitations, it also has its strengths. First, this study investigated the relationship between body mass index, subjective body shape, and suicidal ideation among Korean adults using a nationally representative sample. Therefore, the findings of this study are generalizable to Korean adults. Second, studies on body shapes and suicidal ideation often examined adolescents or younger age groups, but this study examined this association in older age groups as well. Third, this study examined age group differences, and the results can be used to suggest tailored suicide prevention programs according to age groups.

## Conclusion

In conclusion, this study highlights several findings. First, there was an association between body mass index, subjective body shape, and suicidal ideation differed among different age groups. Second, the discrepancy between objective body weight and subjective body shape increased suicidal risk (i.e., healthy weight-fat older adults). Third, people outside the normal range of body shape defined by the society were more likely to have suicidal ideation (i.e., young adults who are overweight-fat, middle-aged adults who are underweight-skinny). More research is needed to explore why the study results differed by age group.

In this study, associations were identified, which can be used as important data for primary suicide prevention. In the future, it would be valuable to examine the above associations longitudinally to identify causal relationships and suggest prevention strategies.

## Data Availability

The datasets used and/or analysed during the current study are available from the corresponding author on reasonable request.

## Abbreviations

BMI: Body mass index
OR: Odds ratio
KCHS: Korean Community Health Survey
CAPI: Computer assisted personal interviewing
PHQ-9: Patient Health Questionnaire-9
M: Mean
SD: Standard deviation
ANOVA: Analysis of variance
